# Supplementary material for: Biogeographic gradients of picoplankton diversity indicate increasing dominance of prokaryotes in warmer Arctic fjords
Source: Commun Biol. 2024 Mar 2;7:256. doi: 10.1038/s42003-024-05946-8 (PMC10908816; doi:10.1038/s42003-024-05946-8)
Supplement: Supplementary file 2 — Supplementary Information [file 42003_2024_5946_MOESM2_ESM.pdf]

Supplementary to:

Biogeographic gradients of picoplankton diversity indicate increasing dominance of prokaryotes in warmer Arctic fjords

Authors

Cora Hörstmann [1,2,3*], Tore Hattermann [4,5], Pauline C. Thomé [6], Pier Luigi Buttigieg [7], Isidora Morel [8], Anya M. Waite [9], Uwe John [1,10]

Affiliations

[1] Alfred Wegener Institute Helmholtz Center for Polar and Marine Research, Am Handelshafen 12, 27570 Bremerhaven, Germany
[2] Aix Marseille Univ, Universite de Toulon, CNRS, IRD, MIO UM 110, 13288, Marseille, France
[3] Turing Center for Living Systems, Aix-Marseille University, 13009 Marseille, France
[4] Norwegian Polar Institute, iC3: Centre for ice, Cryosphere, Carbon and Climate, Framsenteret, Hjalmar Johansens gate 14, 9296 Tromsø, Norway
[5] Complex Systems Group, Department of Mathematics and Statistics, The Arctic University – University of Tromsø, Hansine Hansens veg 18, 9019 Tromsø, Norway
[6] Leibniz Institute of Freshwater Ecology and Inland Fisheries, Müggelseedamm 310, 12587 Berlin, Germany
[7] Helmholtz Metadata Collaboration, GEOMAR, Wischhofstraße 1-3, 24148 Kiel, Germany
[8] Max Planck Institute for Marine Microbiology, Celsiusstraße 1, 28359 Bremen, Germany
[9] Ocean Frontier Institute, Dalhousie University, 1355 Oxford Street, Halifax, Nova Scotia, Canada
[10] Helmholtz Institute for Functional Marine Biodiversity at the University of Oldenburg (HIFMB), Ammerländer Heerstraße 231, 26129 Oldenburg, Germany

*Corresponding author: Cora Hörstmann, cora.hoerstmann@awi.de

Additional information Materials and Methods

Seawater samples were collected during the HE533 expedition between 20.05.2019 and 06.06.2019. Water samples were collected in triplicates (A-C) with Niskin bottles mounted on a Seabird' SBE911+' CTD probe with additional turbidity, oxygen, and fluorescence sensors.

A total of 20 L of seawater at three different depths (3m, DCM which varied between 8-28m, and 40m depth) was pooled and gravity-filtered through 200 µm and 20 µm mesh size sieves, and subsequently filtered through 3 µm and 0.2 µm polycarbonate filters (147 mm diameter, Millipore) using a Millipore Tripod filterholder and a peristaltic pump within a time window of max. 30 min. Warm lysis buffer was added to filters, followed by snap freezing and storage at -80°C until further processing. Picoplankton DNA was extracted using the NucleoSpin® Soil kit (Macherey-Nagel, Germany) following the manufacturer's protocol. For the sample lysis step, a bead beater was used to break up the cells (MagNA Lyser, Roche). As other datasets in this manuscript did not have replicates, we subsampled only the first replicate (A) of the dataset for further analysis. Additionally, we excluded clear outliers from analysis, which were due to a *Crysocromulina* spp. bloom during the HE533 expedition.

DNA:

To extract DNA from the 0.2 µm polycarbonate filters, the Genomic DNA from soil (NucleoSpin® Soil) kit was used following the manufacturer's protocol with a minor modification: the sample lysis was conducted with a bead beater (MagNA Lyser, Roche) for 2 × 30 seconds at 55,000 rpm.

The variable region 4 (V4) of the small subunit ribosomal RNA gene (16S for prokaryotes and 18S for eukaryotes) was used as a molecular marker to determine the taxonomic community composition. Primers were selected in accordance with the Earth Microbiome Project (http://www.earthmicrobiome.org/protocols-and-standards/) using prokaryotic primers (515F - 806R) [1] and eukaryotic primers (TA-Reuk454FWD1 – TAReukREV3) [2] with overhanging Illumina adapters. The library preparation preceding the sequencing followed standard protocols (16S Metagenomic Sequencing Library Preparation, Illumina, Part #15044223 Rev.B; Illumina Technology).

The amplicon libraries were paired-end sequenced on the Illumina MiSeq sequencing platform. The prokaryotic samples were sequenced at the Alfred Wegener Institute in Bremerhaven, Germany, and the eukaryotic samples were sequenced at the Leibniz Institute on Aging (FLI) in Jena, Germany, using 300-bp paired-end sequencing on a MiSeq Sequencer (Illumina) with a MiSeq Reagent Kit v3 (600-cycle). All samples were demultiplexed using bcl2fastq (Illumina) with barcode mismatches set to 1.

Table S1. Tested explanatory variables for prokaryotic and eukaryotic beta diversity distribution.

Taxa	Variable	R^2	F test	p-value
Prokaryotes	temperature (°C)	0.3838	56.679	0.001
	salinity	0.09645	9.714	0.001
	PO_4 (μmol L^{-1})	0.15126	16.217	0.001
	NO_3 (μmol L^{-1})	0.10925	11.161	0.001
	bottom depth (m)	0.02579	2.4086	0.065
	sun altitude (m)	0.04904	4.6929	0.006
Eukaryotes	temperature (°C)	0.18576	20.076	0.001
	salinity	0.08567	8.2456	0.001
	PO_4 (μmol L^{-1})	0.03604	3.2898	0.018
	NO_3 (μmol L^{-1})	0.052	4.8265	0.004
	bottom depth (m)	0.07958	7.6083	0.001
	sun altitude (m)	0.03037	2.7563	0.036
	Fluoresecence	0.0133	1.1863	0.268
	Si (μmol L^{-1})	0.08417	8.0877	0.001

Table S2. Linear regressions of prokaryote and picoeukaryote Aitchinson distance between bioclimatic subzones.

	Eukaryotes	Prokaryotes
temperate - subarctic	value: 0.1201; Adjusted R-squared = 0.2449; p-value: < 2.2e-16	value: 0.150038; Adjusted R-squared: 0.8124; p-value: < 2.2e-16
subarctic - low arctic	value: 0.08754; Adjusted R-squared = 0.3907; p-value: < 2.2e-16	value: 0.089145; Adjusted R-squared: 0.5531 ; p-value: < 2.2e-16
low arctic - high arctic	value: 0.02608; Adjusted R-squared: 0.09815; p-value: 0.01438	value: 0.05004; Adjusted R-squared: 0.1372; p-value: 0.007707

Table S3. Significance levels of alpha diversity measures (Richness and Pielou Evenness) between bioclimatic subzones using two-sample t-test with Bonferroni adjustment of *p*-values.

	Richness		Evenness	
Sites	Two-sample t-test prokaryots (adjusted *p*-value)	Two-sample t-test Eukaryotes (adjusted *p*-value)	Two-sample t-test prokaryots (adjusted *p*-value)	Two-sample t-test Eukaryotes (adjusted *p*-value)
high Arctic – low Arctic	1	1	0.0255	1
high Arctic - subarctic	< 2e-16	0.0027	1	0.47591
high Arctic - temperate	< 2e-16	4.10E-06	1.30E-09	4.00E-06
low Arctic - subarctic	7.20E-10	0.7424	0.0028	1
low Arctic - temperate	6.80E-11	5.40E-07	2.20E-11	0.00099
subarctic - temperate	0.63	5.30E-13	3.00E-08	0.00072

Table S4. Significance levels of differential abundance of functional trophic groups between bioclimatic subzones using two-sample t-test with Bonferroni adjustment of *p*-values.

Sites	autotrophic bacteria (n = 105)	autotrophic archaea (n= 12)	autotrophic eukaryotes (n = 415)	mixotrophic bacteria (n = 39)	mixotrophic eukaryotes (n = 149)	heterotrophic bacteria (n = 888)	heterotrophic eukaryotes (n = 921)
Arctic (n = 12) - subarctic (n = 33)	1.2e-06	0.00020	4.7e-05	0.0039	0.00013	1.2e-06	6.0e-06
Arctic (n = 12) - temperate (n = 17)	0.03360	0.56091	0.004	1.0000	0.06422	0.02310	0.33
subarctic (n = 33) - temperate (n = 17)	0.00016	0.00021	0.296	2.4e-05	0.01853	0.00031	5.7e-06

Table S5. Cross-links to dataset publications and data availability.

Expedition	Region	Methods (Nutrients/ DNA extraction/ Sampling)	Physical oceanography (PANGAEA)	Nutrients (PANGAEA/ Supplement to publications)	DNA sequences (ENA)
HE533	North.Norway	This MS Supplementary	https://doi.org/10.1594/PANGAEA.903511	https://doi.org/10.1594/PANGAEA.928451	PRJEB50059
HE431	North.Norway/ South.Norway/ Sweden	https://doi.org/10.3390/microorganisms8040567	https://doi.org/10.1594/PANGAEA.863438	https://doi.org/10.1594/PANGAEA.928449	PRJEB50592
HE492	Svalbard	https://doi.org/10.1525/elementa.2021.00117	https://doi.org/10.1594/PANGAEA.881306	https://doi.org/10.1594/PANGAEA.928449 (chlorophyll only)	PRJEB49358
MSM56	Svalbard/ East.Greenland	https://doi.org/10.3389/fmars.2019.00412	https://doi.org/10.1594/PANGAEA.871015	https://doi.org/10.3389/fmars.2019.00412	PRJEB50596
MSM21/3	Iceland/ West.Greenland	https://doi.org/10.1016/j.dsr.2016.11.002	https://doi.org/10.1594/PANGAEA.819731	https://doi.org/10.1594/PANGAEA.897293	PRJEB50593

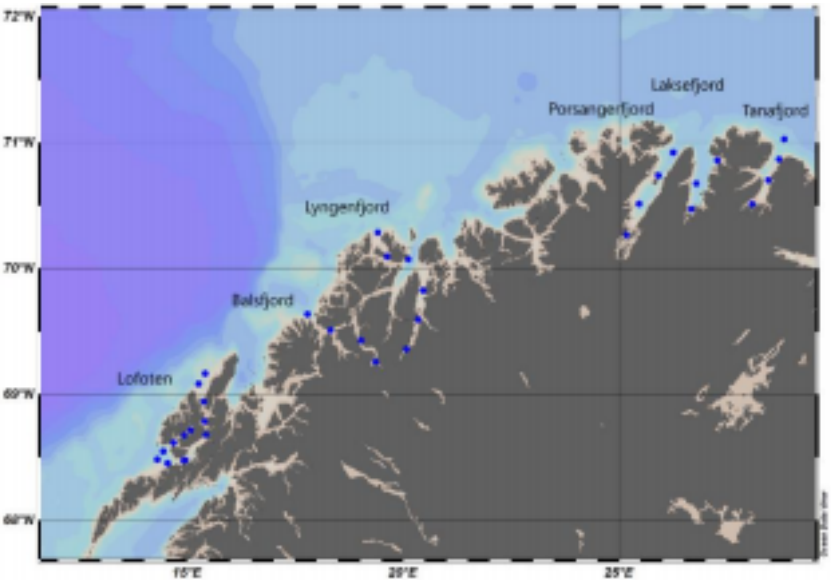

Figure S1. Sample locations in northern Norway. Individual fjord names are indicated in the map. Map created with Ocean Data View[3].

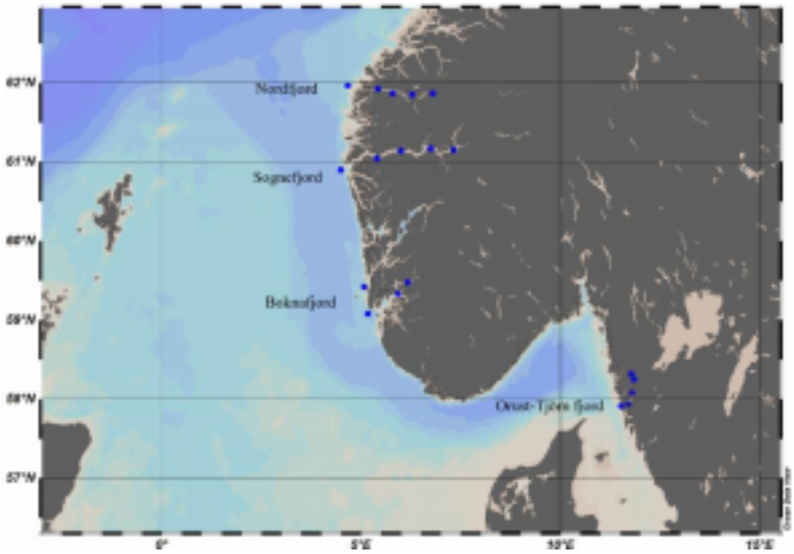

Figure S2. Sample locations in southern Norway and Sweden. Individual fjord names are indicated in the map. Map created with Ocean Data View[3].

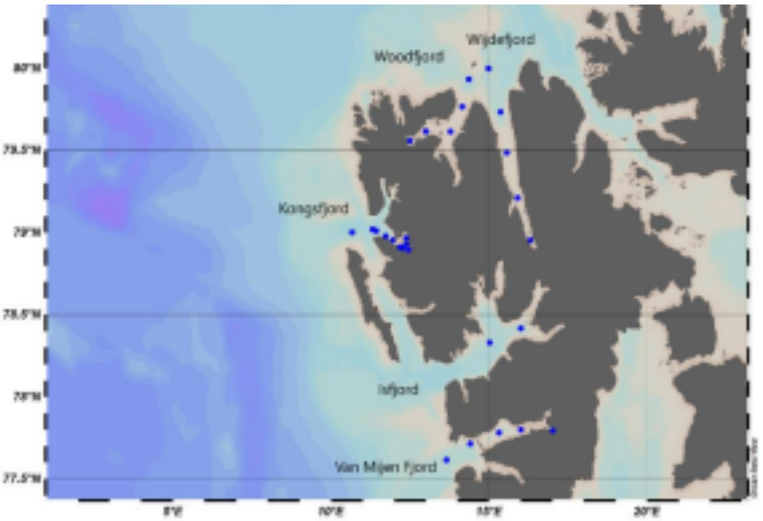

Figure S3. Sample locations in Svalbard. Individual fjord names are indicated in the map. Map created with Ocean Data View[3]

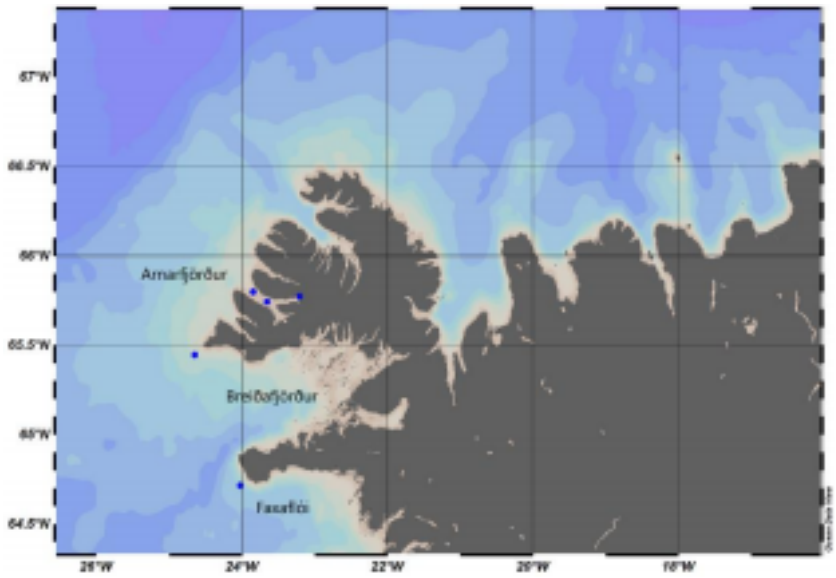

Figure S4. Sample locations in Iceland. Individual fjord names are indicated in the map. Map created with Ocean Data View[3]

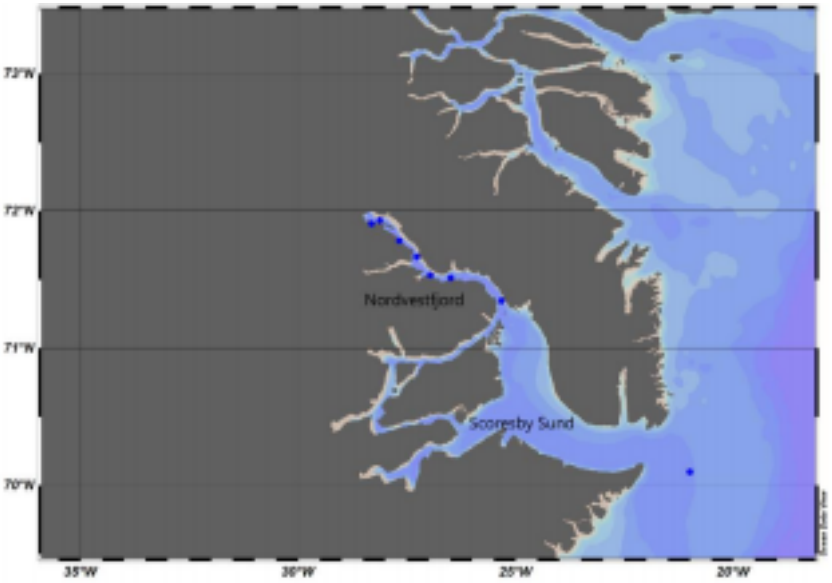

Figure S5. Sample locations in East Greenland. Individual fjord names are indicated in the map. Map created with Ocean Data View[3].

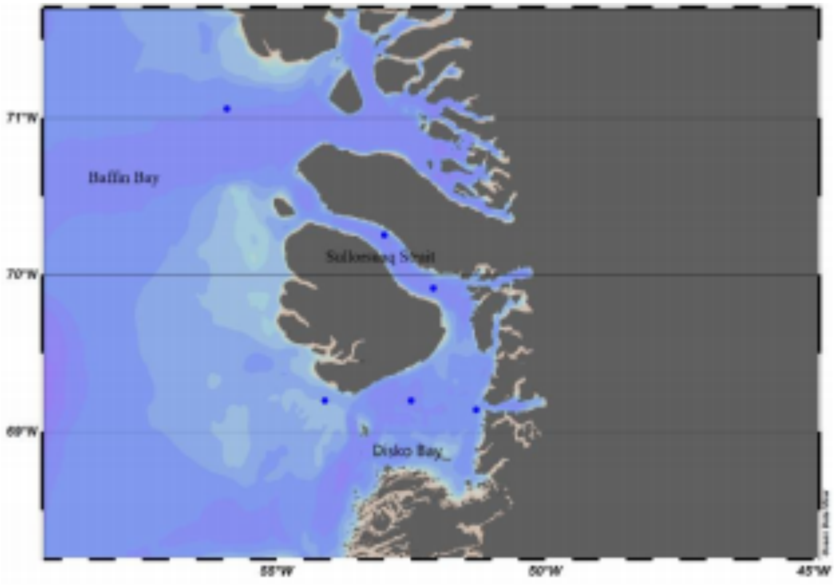

Figure S6. Sample locations in West Greenland. Individual fjord names are indicated in the map. Map created with Ocean Data View[3].

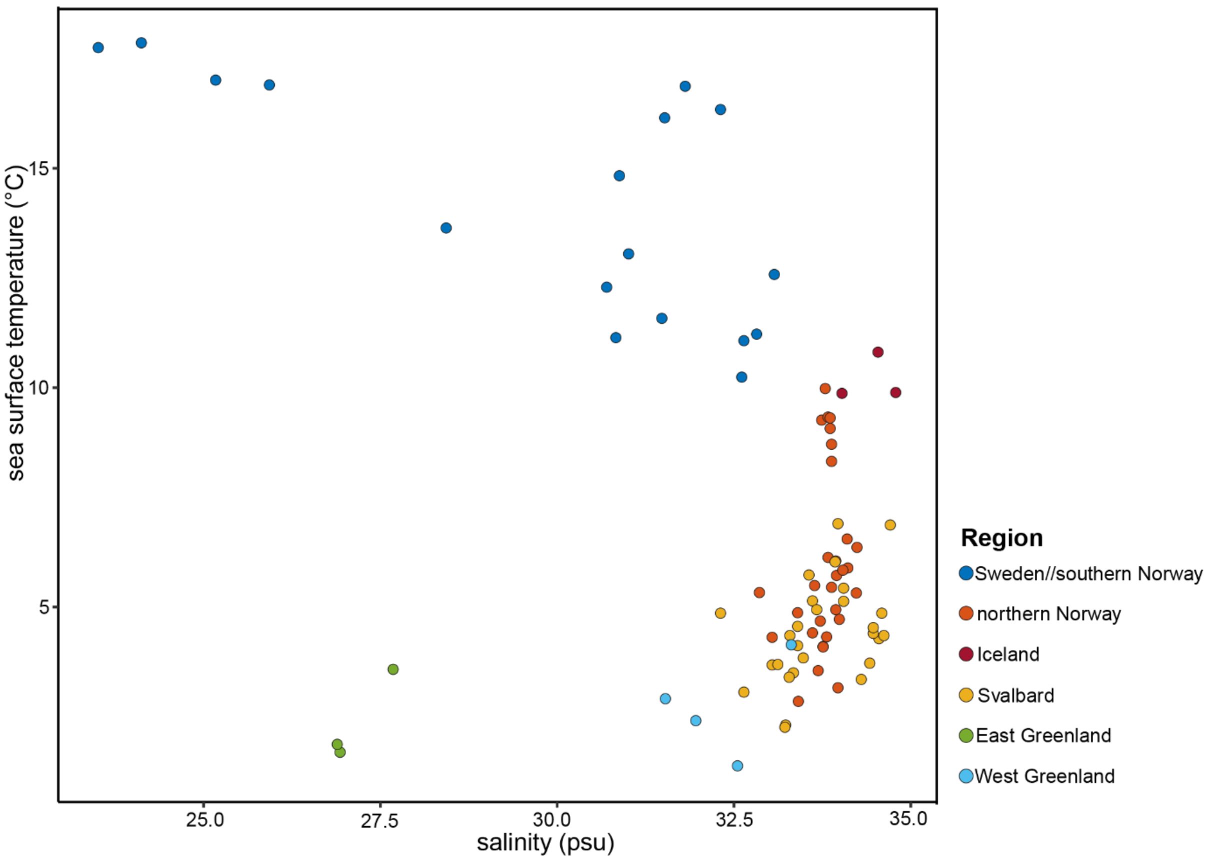

Figure S7. Temperature – salinity plot of all sites from surface water samples. Sites are color-coded according to geographic region.

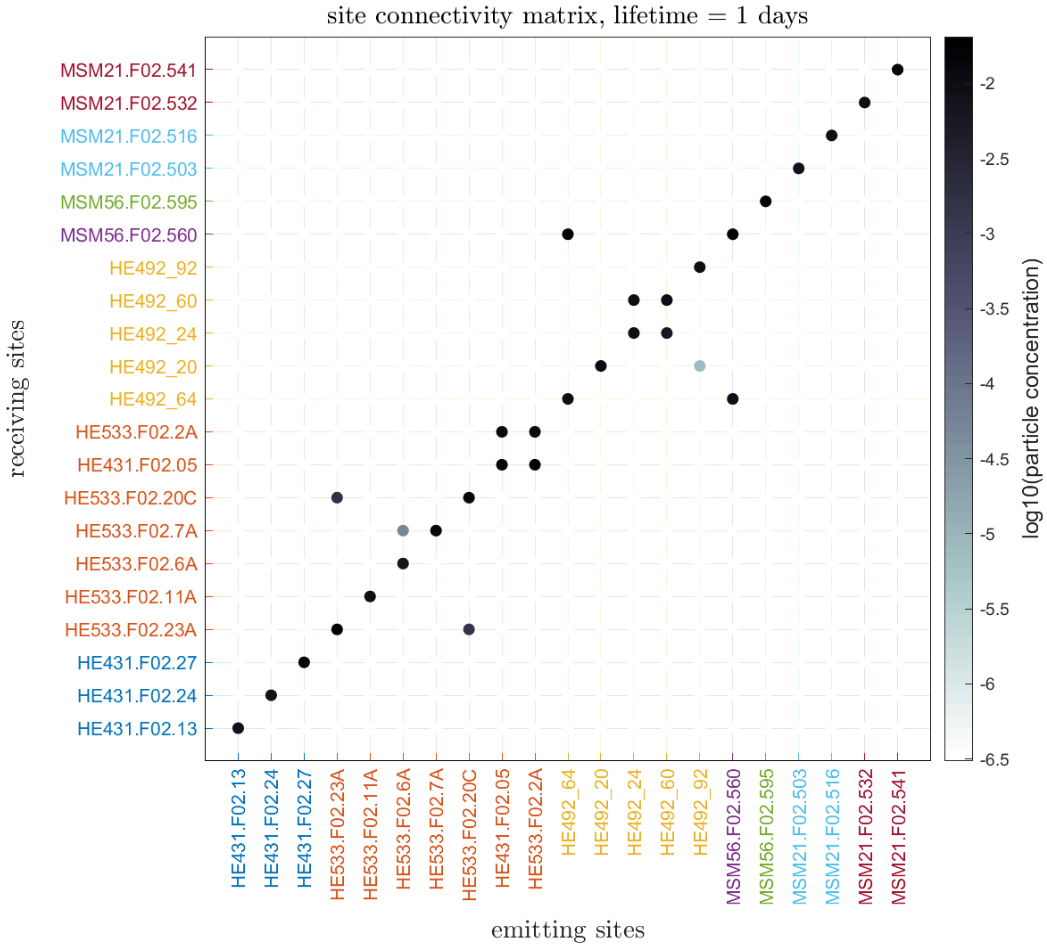

Figure S8. Connectivity matrix of synthetic numerical drifters that are 1 day old.

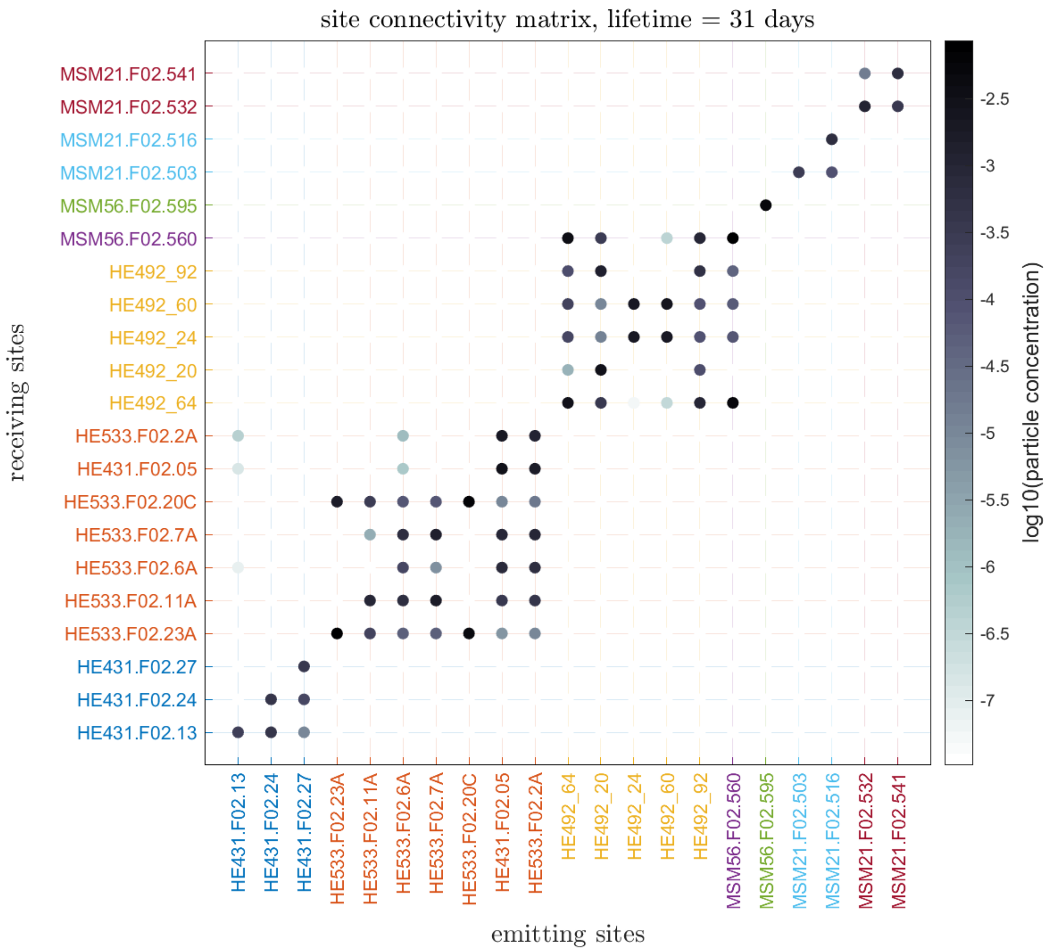

Figure S9. Connectivity matrix of synthetic numerical drifter concentration after 1 month (31 days)

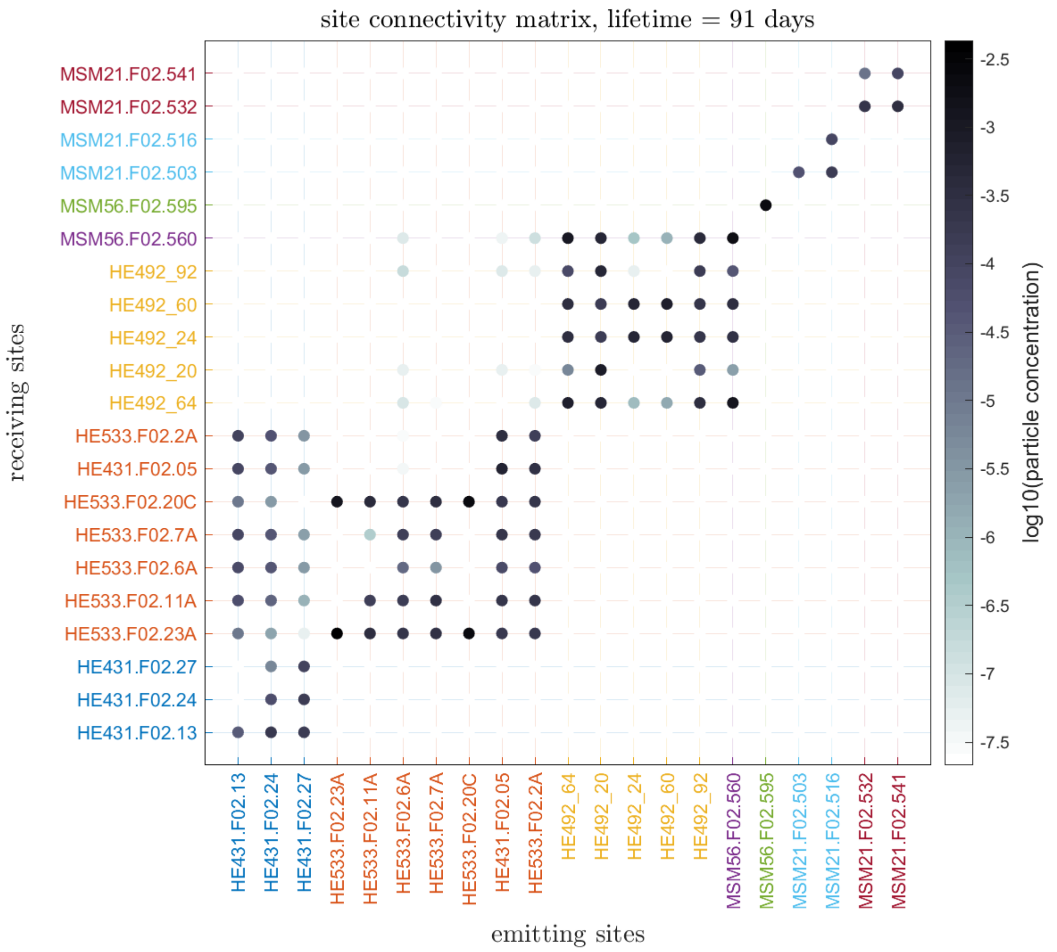

Figure S10. Connectivity matrix of synthetic numerical drifter concentration after 3 months (91 days)

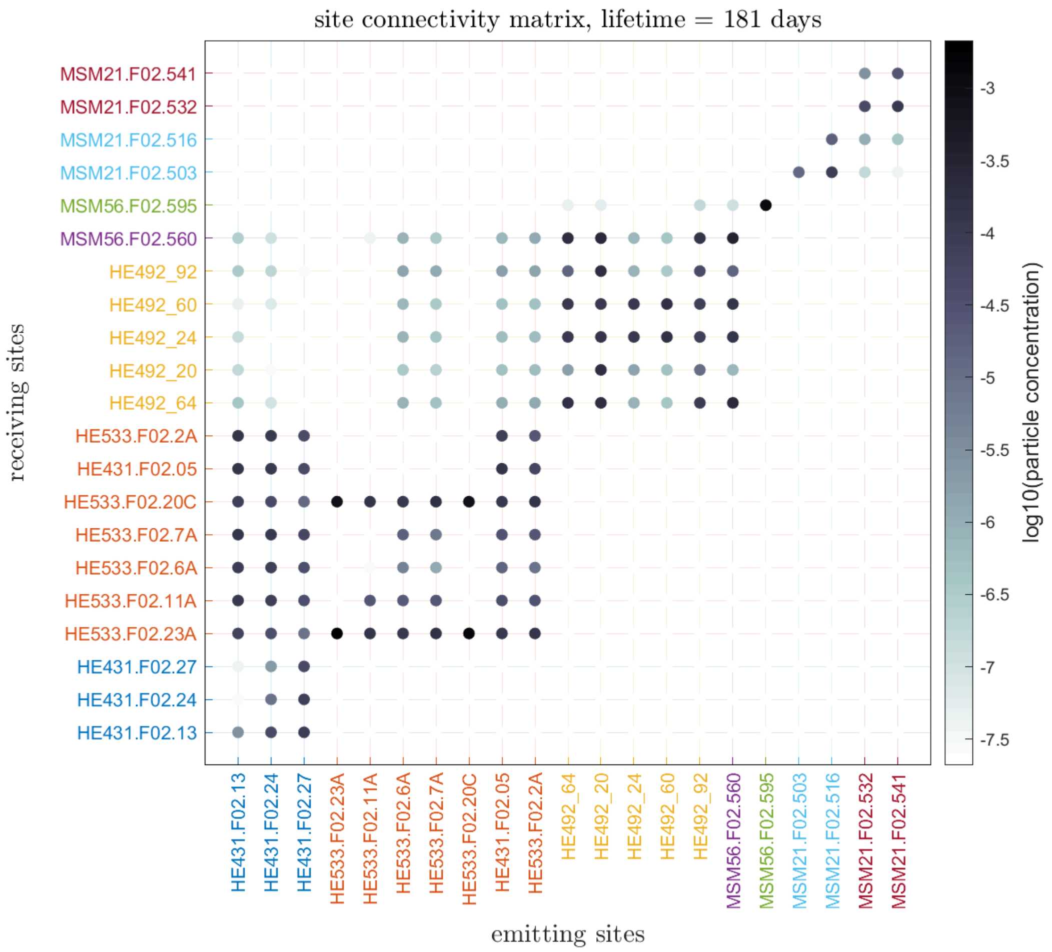

Figure S11. Connectivity matrix of synthetic numerical drifter concentration after 6 months (181 days)

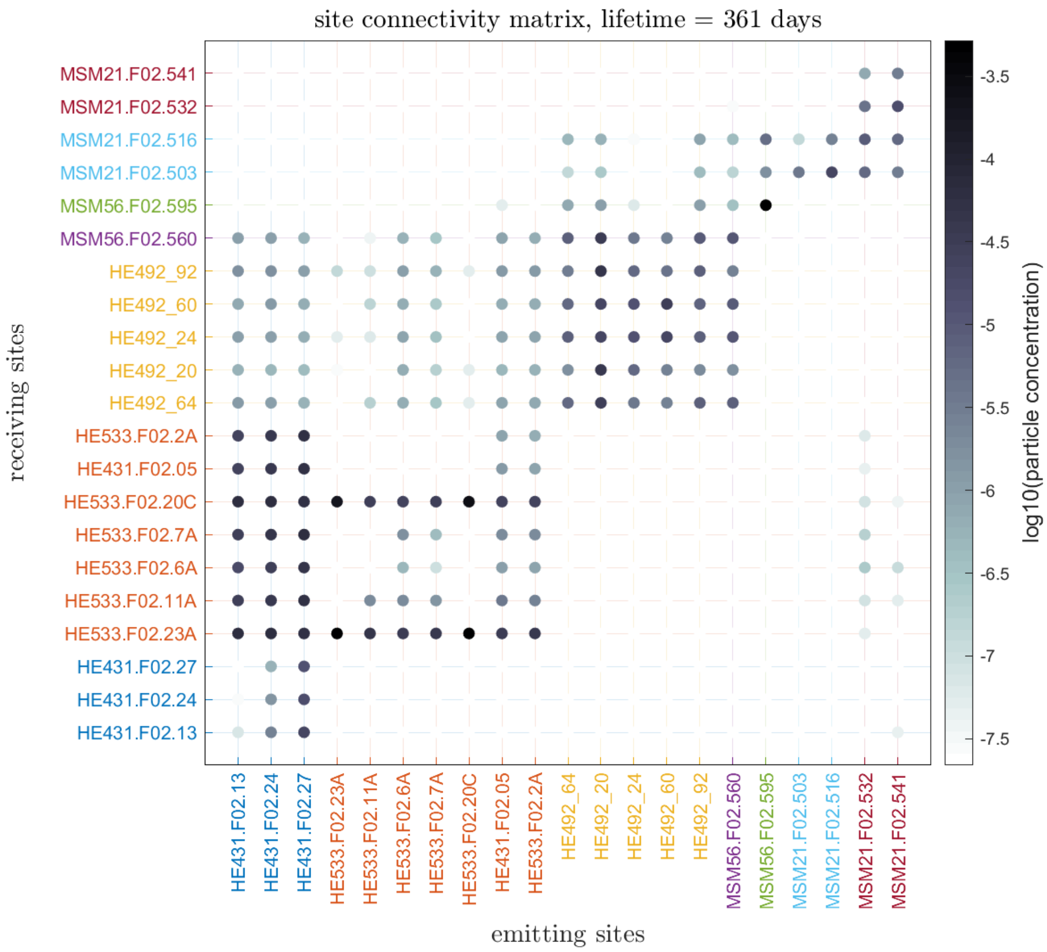

Figure S12. Connectivity matrix of synthetic numerical drifter concentration after 1 year (361 days).

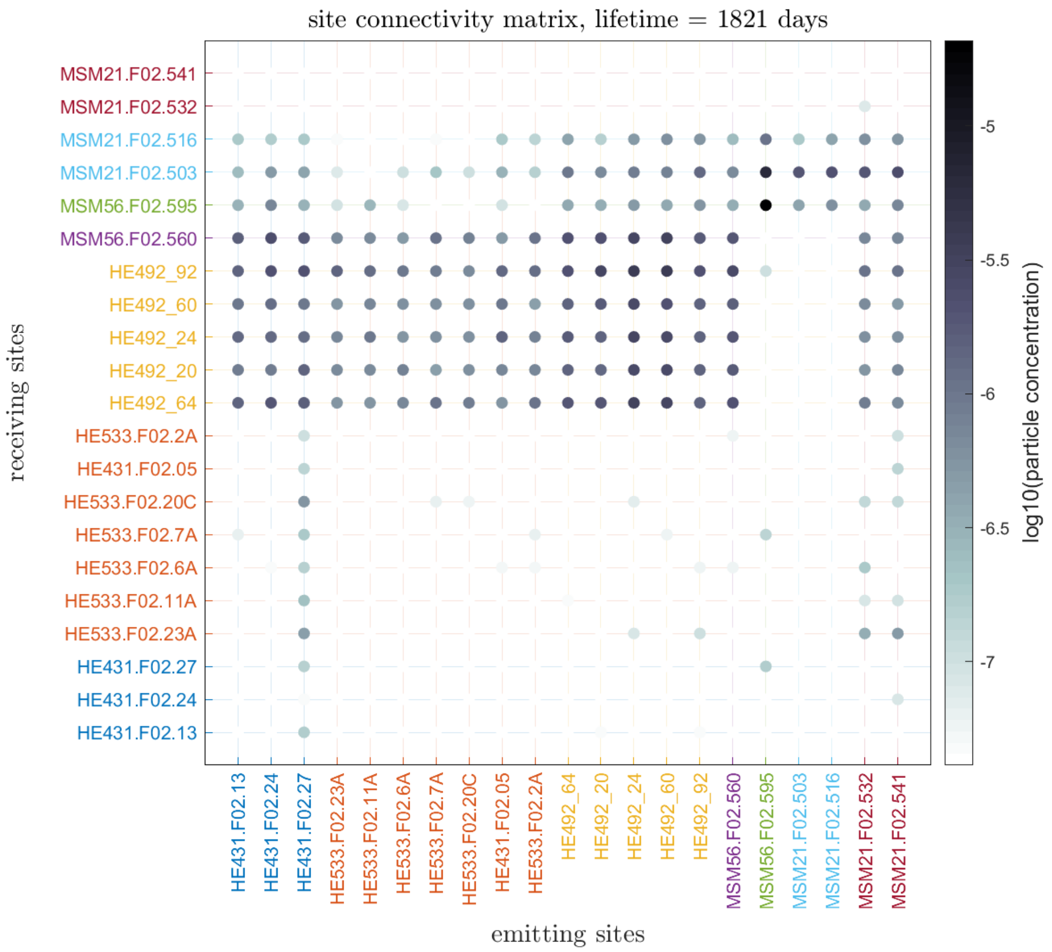

Figure S13. Connectivity matrix of synthetic numerical drifter concentration after 5 years (1821 days).

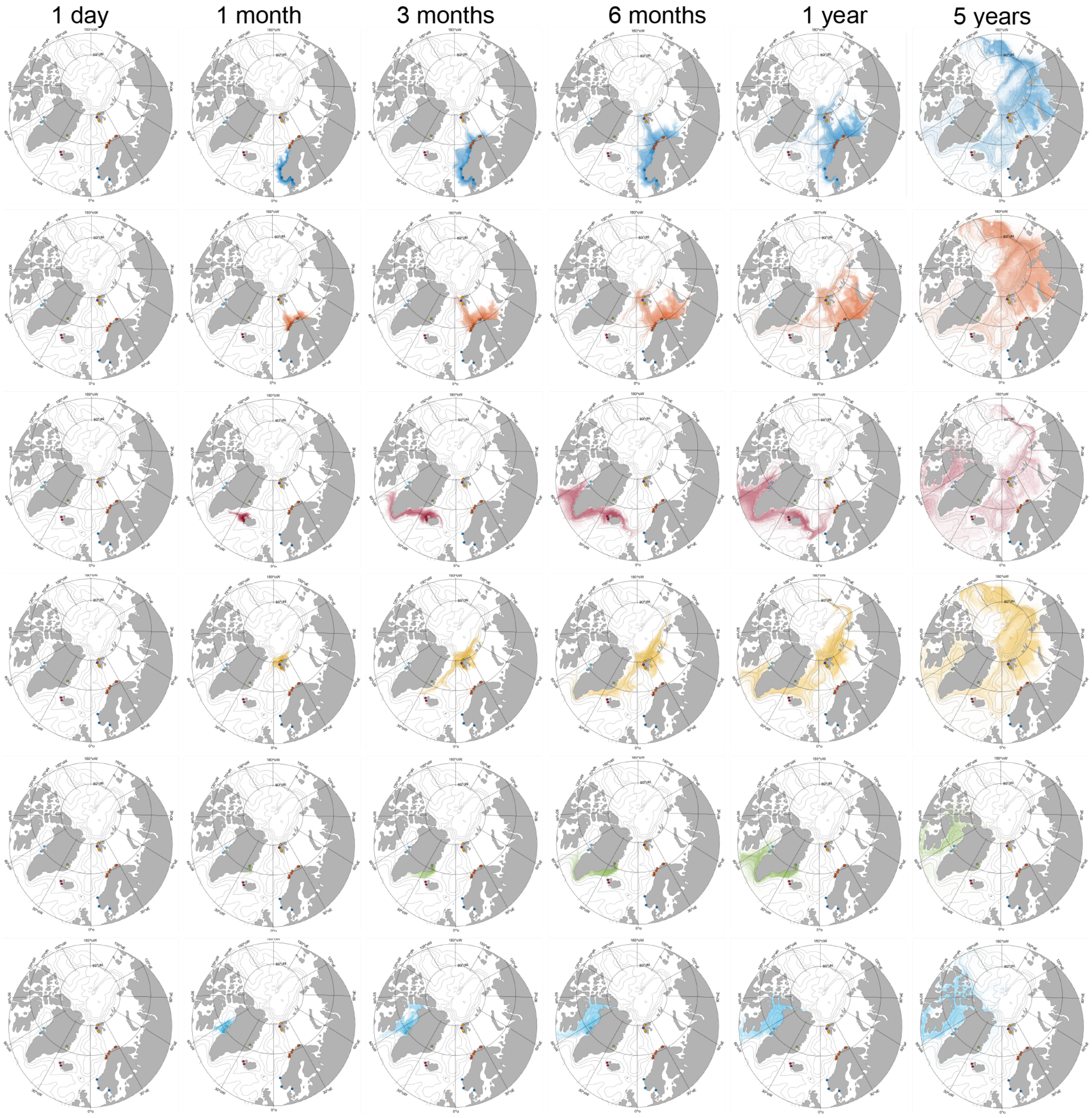

Figure S14. Synthetic particle dispersal of individual regions after 1 day, 1 month, 3 months, six months, 1 year, and 5 years.

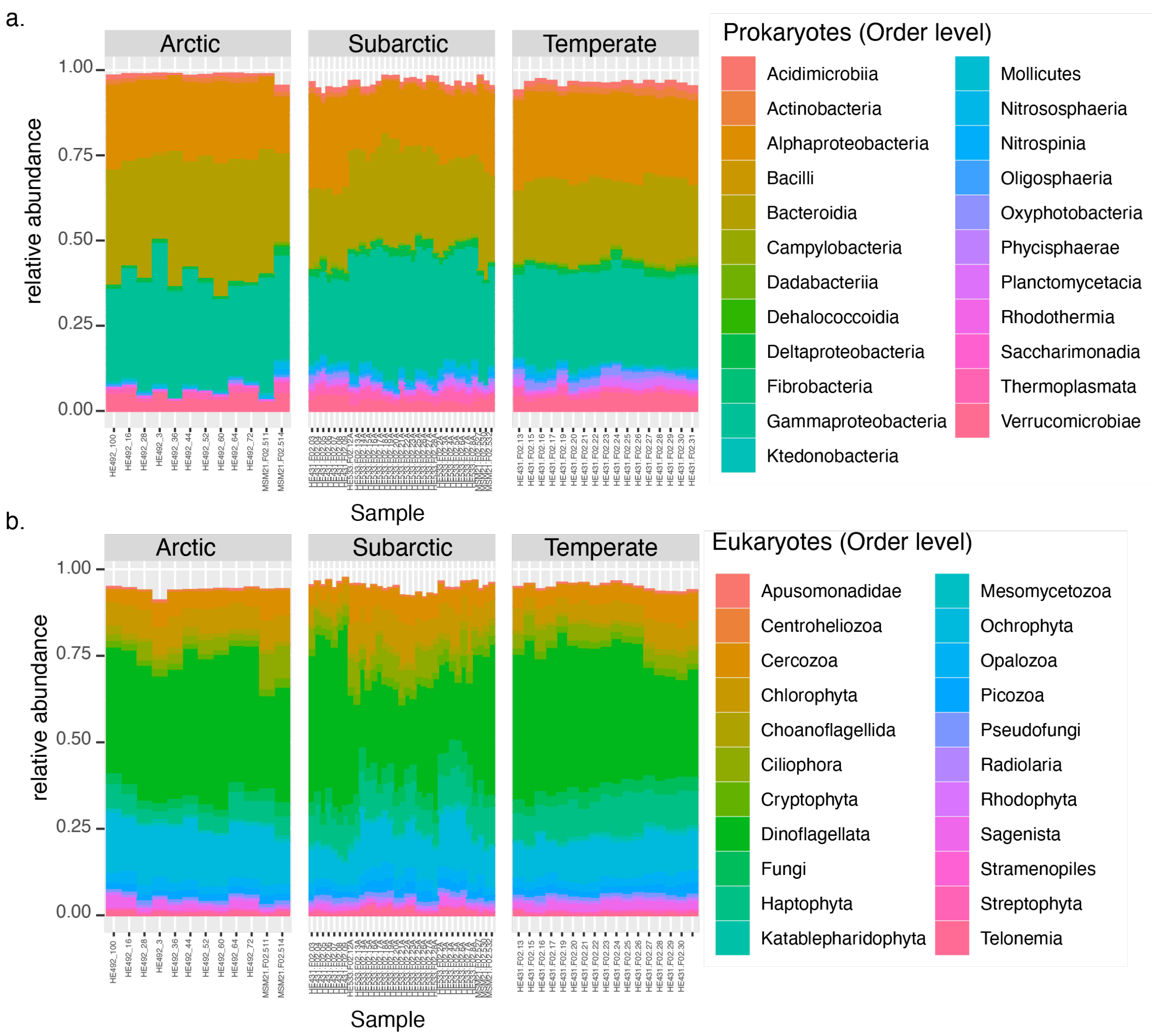

Figure S15. Relative abundances of prokaryotic (a) and picoeukaryotic (b) taxa at the Order level.

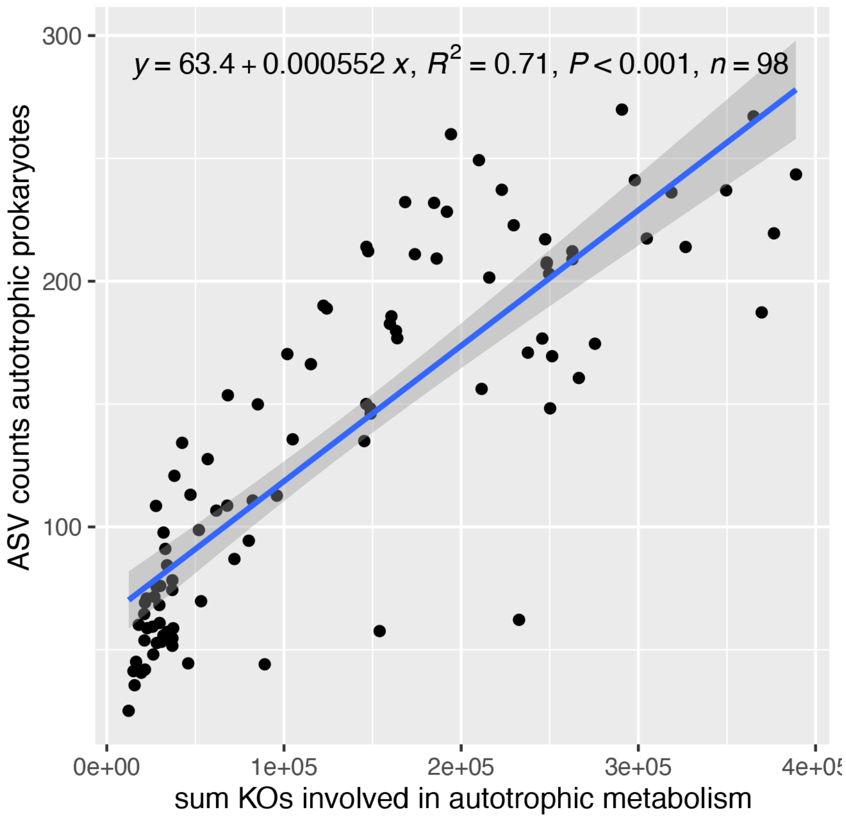

Figure S16. ASV abundance of prokaryotes annotated as “autotroph” based on literature research mapped against functional inference derived from PICRUST2 including all KOs involved in autotrophy (see Table S4 for details).

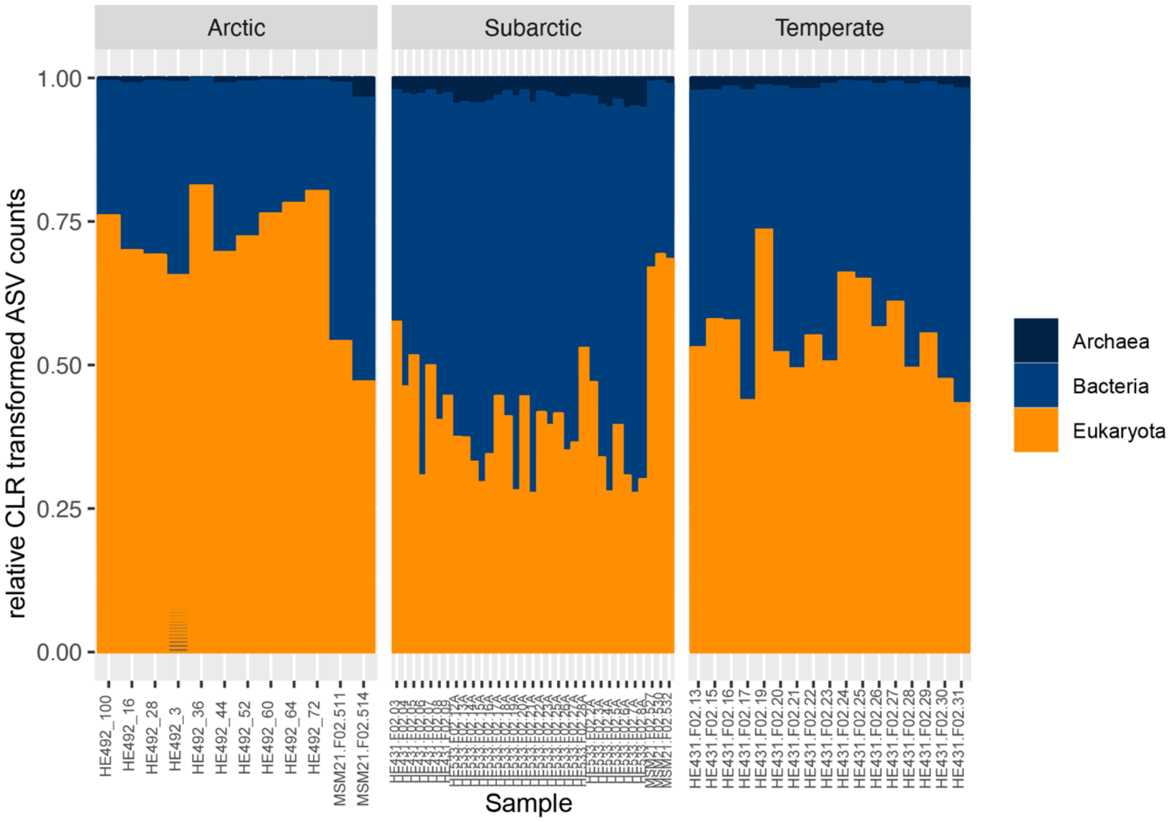

Figure S17. Relative proportion of bacteria, archaea and eukaryotes in each station separated between Arctic, subarctic and temperate regions.

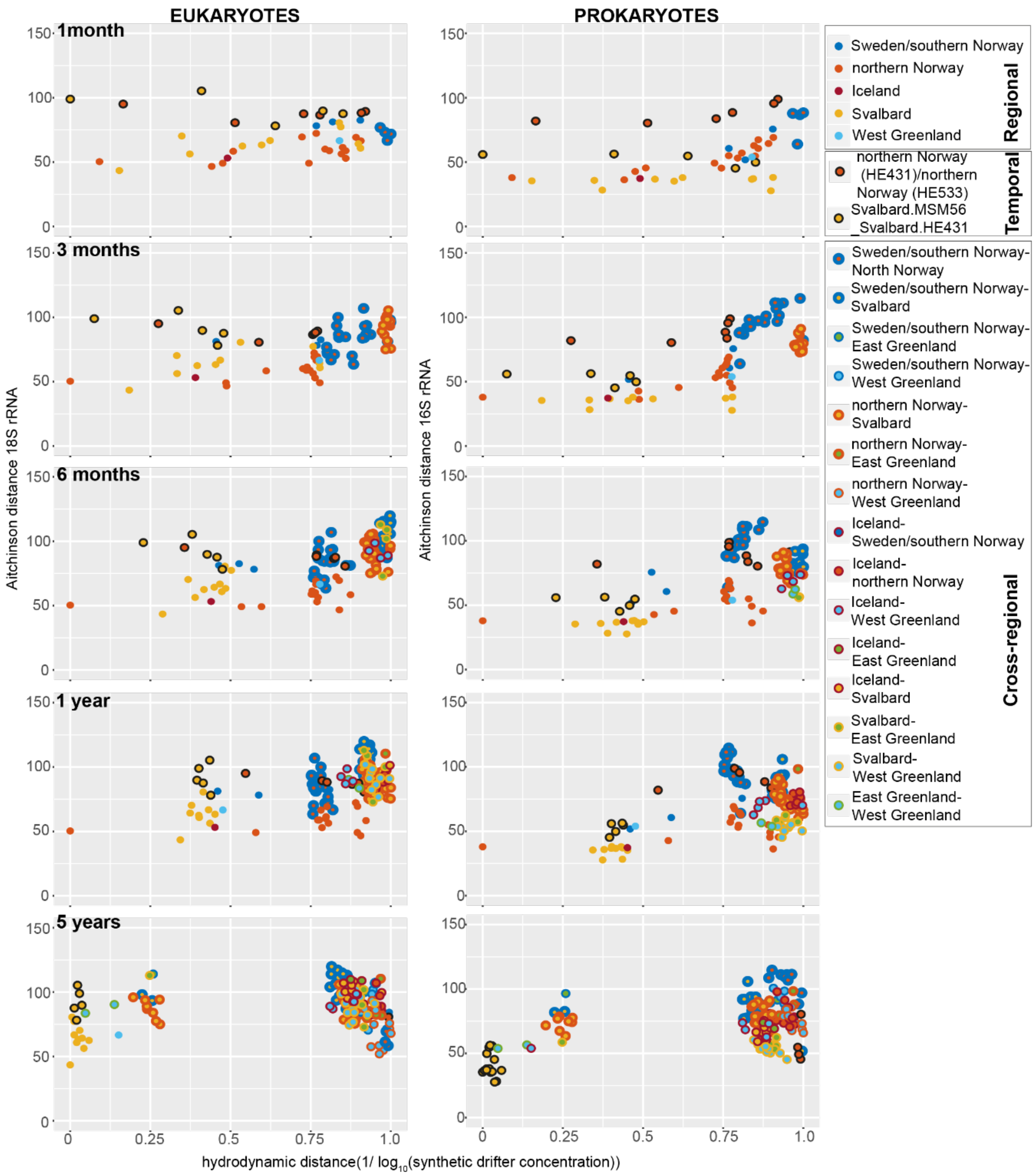

Figure S18. Microbial beta diversity distance (Aitchinson distance) based on 16S rRNA (prokaryotes) and 18S rRNA (eukaryotes) sequences analyses against hydrodynamic distance based on the inverse of the normalized log_{10} synthetic particle concentration.

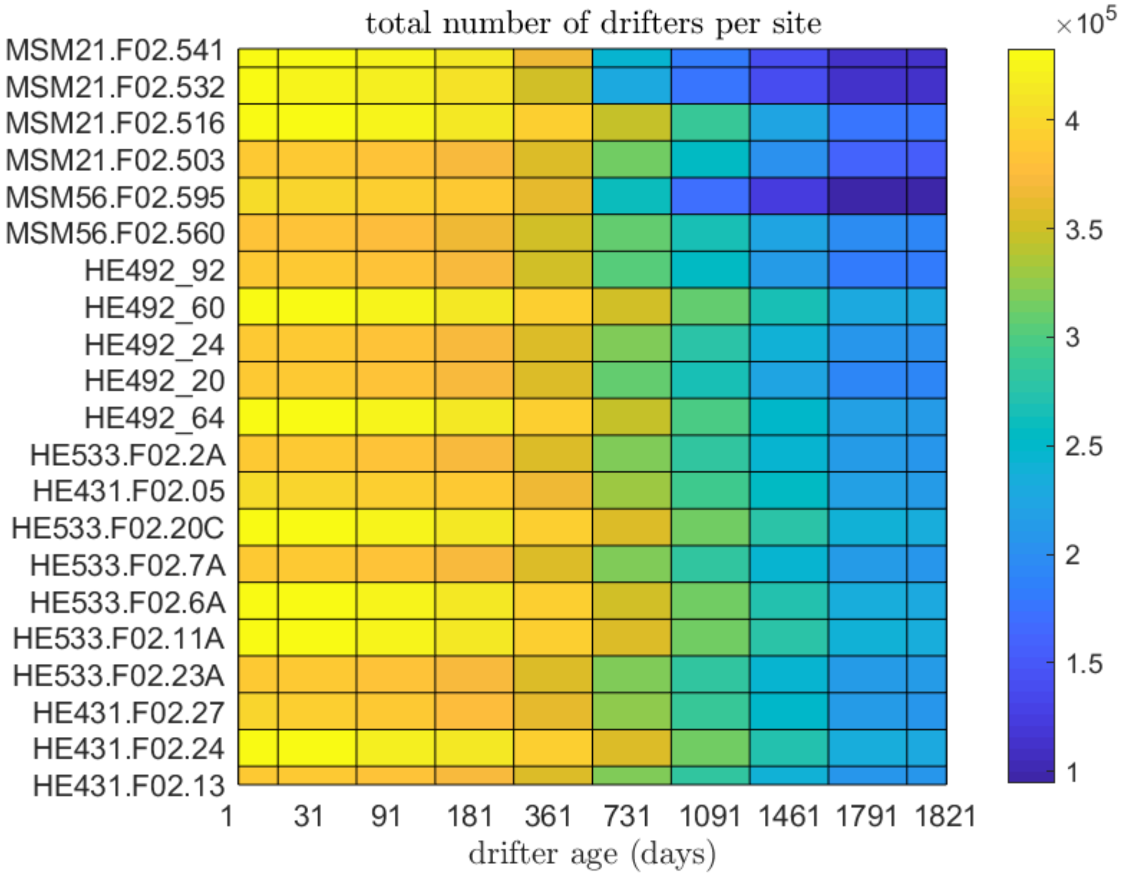

Figure S19. Total number of numerical synthetic drifters per site and for individual temporal bins.

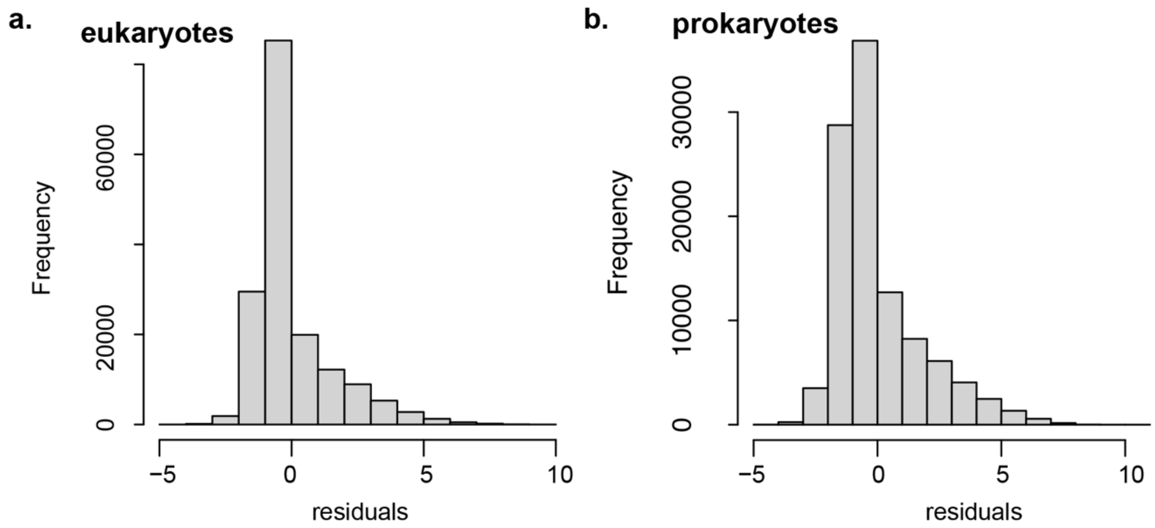

Figure S20. Residuals of both a. eukaryotic and b. prokaryotic RDA are normally distributed.

Supplementary References

1. Caporaso, J. G. *et al.* Moving pictures of the human microbiome. *Genome Biol.* **36**, 50–80 (2016).
2. Stoeck, T., Bass, D., Nebel, M., Christen, R. & Meredith, D. Multiple marker parallel tag environmental DNA sequencing reveals a highly complex eukaryotic community in marine anoxic water. *Mol. Ecol.* **19**, 21–31 (2010).
3. Schlitzer, R. Ocean Data View v5.6.3. (2016).
