# Supplementary material for: Biogeographic gradients of picoplankton diversity indicate increasing dominance of prokaryotes in warmer Arctic fjords
Source: Commun Biol. 2024 Mar 2;7:256. doi: 10.1038/s42003-024-05946-8 (PMC10908816; doi:10.1038/s42003-024-05946-8)
Supplement: Supplementary file 3 — Description of Additional Supplementary Files [file 42003_2024_5946_MOESM3_ESM.pdf]

Description of Additional Supplementary Files

File name: Supplementary Data 1

Description: Sampling stations including time, location, dissolved inorganic nutrient concentrations, calculated sun elevation angle, bottom depth ranges, as well as grouping into bioclimatic regions following the classification by the Circumpolar Arctic Vegetation Mapping Project (https://www.arcticcentre.org/EN/arcticregio n/Maps/definitions), geographic location, individual fjords and according to the presence of marine-terminating glaciers.

File name: Supplementary Data 2

Description: Literature research and functional trophic assignment of prokaryotic and picoeukaryotic ASVs.

File name: Supplementary Data 3

Description: Taxonomic classification of prokaryotic ASVs using the Silva v123 database

File name: Supplementary Data 4

Description: Taxonomic classification of eukaryotic ASVs using the pr2 v4.12 database

File name: Supplementary Data 5

Description: KO numbers profiled from corresponding shotgun metagenomes (MGS)
