# Supplementary material for: Biogeographic gradients of picoplankton diversity indicate increasing dominance of prokaryotes in warmer Arctic fjords
Source: Commun Biol. 2024 Mar 2;7:256. doi: 10.1038/s42003-024-05946-8 (PMC10908816; doi:10.1038/s42003-024-05946-8)
Supplement: Supplementary file 9 — Reporting Summary [file 42003_2024_5946_MOESM9_ESM.pdf]

nature portfolio

Corresponding author(s): Cora Hörstmann

Last updated by author(s): 01/02/2024

# Reporting Summary

Nature Portfolio wishes to improve the reproducibility of the work that we publish. This form provides structure for consistency and transparency in reporting. For further information on Nature Portfolio policies, see our Editorial Policies and the Editorial Policy Checklist.

## Statistics

For all statistical analyses, confirm that the following items are present in the figure legend, table legend, main text, or Methods section.

| n/a | Confirmed | |
|---|---|---|
| ☐ | ☒ | The exact sample size (*n*) for each experimental group/condition, given as a discrete number and unit of measurement |
| ☐ | ☒ | A statement on whether measurements were taken from distinct samples or whether the same sample was measured repeatedly |
| ☐ | ☒ | The statistical test(s) used AND whether they are one- or two-sided<br>*Only common tests should be described solely by name; describe more complex techniques in the Methods section.* |
| ☒ | ☐ | A description of all covariates tested |
| ☐ | ☒ | A description of any assumptions or corrections, such as tests of normality and adjustment for multiple comparisons |
| ☐ | ☒ | A full description of the statistical parameters including central tendency (e.g. means) or other basic estimates (e.g. regression coefficient) AND variation (e.g. standard deviation) or associated estimates of uncertainty (e.g. confidence intervals) |
| ☐ | ☒ | For null hypothesis testing, the test statistic (e.g. *F*, *t*, *r*) with confidence intervals, effect sizes, degrees of freedom and *P* value noted<br>*Give P values as exact values whenever suitable.* |
| ☒ | ☐ | For Bayesian analysis, information on the choice of priors and Markov chain Monte Carlo settings |
| ☒ | ☐ | For hierarchical and complex designs, identification of the appropriate level for tests and full reporting of outcomes |
| ☐ | ☒ | Estimates of effect sizes (e.g. Cohen's *d*, Pearson's *r*), indicating how they were calculated |

*Our web collection on statistics for biologists contains articles on many of the points above.*

## Software and code

Policy information about availability of computer code

| | |
|---|---|
| Data collection | n/A |
| Data analysis | ocean synthetic particle drift model: metROMS and ROMS (cited in MM section)<br>all statistical analysis: R v4.2.2 and RStudio v2022.12.0+353 with the following packages: (code available on gitHub)<br>dplyr v1.0.10; ggplot2 v3.4.0; ggords v1.0; gridExtra v3.2; iNEXT v3.0.0; tidyverse v1.3.2; reshape2 v1.4.4; vegan 2.6.4<br>suncalc v0.5.1; zCompositions v1.4.0.1; metagMisc v.0.0.4; rcompanion v2.4.21; phyloseq 1.42.0; fantaxic 0.2.0 |

For manuscripts utilizing custom algorithms or software that are central to the research but not yet described in published literature, software must be made available to editors and reviewers. We strongly encourage code deposition in a community repository (e.g. GitHub). See the Nature Portfolio guidelines for submitting code & software for further information.

## Data

Policy information about availability of data

All manuscripts must include a data availability statement. This statement should provide the following information, where applicable:

- Accession codes, unique identifiers, or web links for publicly available datasets
- A description of any restrictions on data availability
- For clinical datasets or third party data, please ensure that the statement adheres to our policy

Sequence data for this study have been deposited in the European Nucleotide Archive (ENA) at EMBL- EBI under accession numbers
MSM56 PRJEB50596 (MSM56), PRJEB50593 (MS21-3), PRJEB50592 (HE431), PRJEB49358 (HE492), PRJEB50059 (HE533),
using the brokerage service of the German Federation for Biological Data [GFBio 63] in compliance with the Minimal Information about any (X)

Sequence (MIxS) standard. Contextual metadata is currently in submission to PANGAEA. We will make sure that the data is available latest with publication of this manuscript.

# Field-specific reporting

Please select the one below that is the best fit for your research. If you are not sure, read the appropriate sections before making your selection.

☐ Life sciences ☐ Behavioural & social sciences ☒ Ecological, evolutionary & environmental sciences

For a reference copy of the document with all sections, see nature.com/documents/nr-reporting-summary-flat.pdf

# Ecological, evolutionary & environmental sciences study design

All studies must disclose on these points even when the disclosure is negative.

**Study description**

This study tested how picoplankton communities (prokaryotes and picoeukaryotes) shift trough oceanographic transport between different fjord systems while being locally restricted by environmental conditions.

**Research sample**

93 samples were analyzed from five research cruises in Arctic, sub-Arctic and temperate fjords during spring and summer between 2014–2019. Sample metadata is described in Table S1 and links to individual datasets compiled in Table S2. Samples were selected to match the following criteria:
1) samples were located in Arctic, subarctic and temperate fjord system of the Atlantic and Greenlandic sector of the Atlantic and Arctic Ocean
2) picoplanktonic size fraction
3) Summer in the northern Hemisphere
The highly conserved 16S rRNA and 18S rRNA genes are widely used for prokaryotic and eukaryotic sequence identification. Prokaryotic primers (515F–806R) and eukaryotic primers (TA-Reuk454FWD1–TAReukREV3) for all samples were selected in accordance with the Earth Microbiome Project (http://www.earthmicrobiome.org/protocols-and-standards/). All samples were sequenced on a Illumina MiSeq sequencer.

**Sampling strategy**

sample size was predetermined. All samples from Arctic and sub-Arctic fjords (n = 93) from surface water, size-fractionation between 0.2 and 3um were used in our analyses. Oceanographic connectivity between sites was modelled using metROMS (https://doi.org/10.5281/zenodo.290667), which couples the state-of-the-art Regional Ocean Modeling System (ROMS, http://myroms.org), a free-surface, hydrostatic, primitive equation ocean general circulation model.

**Data collection**

Data was previously collected as part of five distinct research expeditions by multiple scientists. Cora Hörstmann and Uwe John compiled available data for the meta-analysis in this study.

**Timing and spatial scale**

Sampling data was used from research cruises between 2014 and 2019. 93 samples were taken from 21 different Arctic and sub-Arctic fjords (3-8 samples per fjord). Synthetic float modelling the oceanographic connectvitiy between 23 sites was run with a horizontal resolution of 4 × 4 km in an orthogonal, curvilinear grid covering the entire Arctic Mediterranean over 2005¬–2017. The initial state and boundary conditions were derived from monthly-averaged global reanalyses and additional forcing along its open boundaries using the global TPXO tidal model. Atmospheric forcing was conducted via 6-h ERA-Interim reanalysis. Output contained velocity fields in 32 terrains following vertical layers, and a temporal resolution of 24 h.

**Data exclusions**

Replicates B and C from research expedition H533 were excluded as all other research expedition did not have any replicates per site.

**Reproducibility**

Bioinformatic analyses are described in the materials and methods and publicly available (https://doi.org/10.5281/zenodo.7827904)
All data are publicly available

**Randomization**

Data was grouped according to 1) geographic location, 2) individual fjords, 3) presence of marine-terminating glaciers. co-variation was tested and discussed.

**Blinding**

Data blinding was not applicable as this study was not a clinical study but rather a mechanistic ecological study

Did the study involve field work? ☒ Yes ☐ No

## Field work, collection and transport

**Field conditions**

This oceanographic study compiles multiple datasets from five distinct research expeditions. All expeditions took place in spring and summer.

**Location**

All samples are surface water samples from 0-40m depth. All data is from the Atlantic sector of the Arctic Ocean (Latitude: 61-79°N, and -55 - 27°E)

**Access & import/export**

all data fall under the Nagoya protocol.

**Disturbance**

n/A

# Reporting for specific materials, systems and methods

We require information from authors about some types of materials, experimental systems and methods used in many studies. Here, indicate whether each material, system or method listed is relevant to your study. If you are not sure if a list item applies to your research, read the appropriate section before selecting a response.

## Materials & experimental systems

| n/a | Involved in the study |
|---|---|
| ☒ | ☐ Antibodies |
| ☒ | ☐ Eukaryotic cell lines |
| ☒ | ☐ Palaeontology and archaeology |
| ☒ | ☐ Animals and other organisms |
| ☒ | ☐ Human research participants |
| ☒ | ☐ Clinical data |
| ☒ | ☐ Dual use research of concern |

## Methods

| n/a | Involved in the study |
|---|---|
| ☒ | ☐ ChIP-seq |
| ☒ | ☐ Flow cytometry |
| ☒ | ☐ MRI-based neuroimaging |
